# A review of Mpox: Biological characteristics, epidemiology, clinical features, diagnosis, treatment, and prevention strategies

**DOI:** 10.1002/EXP.20230112

**Published:** 2024-10-08

**Authors:** Lin Jiang, Ailan Xu, Lin Guan, Yong Tang, Guangshuai Chai, Junya Feng, Yueqi Wu, Maochen Li, Chuxie Zhang, Xiaojing Liu, Xiaolong Xu, Qingquan Liu, Lihua Song, Yigang Tong, Renald Blundell, Huahao Fan

**Affiliations:** ^1^ College of Life Science and Technology Beijing University of Chemical Technology Beijing China; ^2^ Department of Respiratory and Critical Care Medicine Qianfo Mountain Hospital of Shandong University Jinan Shandong China; ^3^ Department of Biomedical Engineering The Chinese University of Hong Kong Hong Kong China; ^4^ Institute of Photomedicine Shanghai Skin Disease Hospital Shanghai China; ^5^ Beijing Hospital of Traditional Chinese Medicine Capital Medical University Beijing China; ^6^ Department of Physiology and Biochemistry, Faculty of Medicine and Surgery University of Malta Imsida Malta; ^7^ School of Life Sciences Tianjin University Tianjin China

**Keywords:** monkeypox, Mpox, MPXV, *Orthopoxvirus*, viral outbreak

## Abstract

The outbreak of monkeypox virus (MPXV) was declared a Public Health Emergency of International Concern (PHEIC) by the World Health Organization (WHO), and the zoonotic disease caused by viral infection was renamed as “Mpox” on November 28, 2022. Currently, there is no approved vaccine or specific antiviral treatment for Mpox, and a main preventive strategy against MPXV infection remains the smallpox vaccine. Although there was an emergency use authorization (EUA) of Brincidofovir and Tecovirimat for the clinical treatment of clade II Mpox, while Tecovirimat failed to reduce the duration of Mpox lesions among patients infected with clade I Mpox in the Democratic Republic of the Congo (DRC). Therefore, it is still an urgent need to develop an effective medication. This review aims to enhance the understanding of Mpox and contribute to its prevention and treatment strategies, it provides a systemic introduction of the biological and epidemiological characteristics of MPXV, the clinical feature and diagnosis of Mpox, as well as treatment and prevention strategies, which will improve the comprehension about MPXV and offer potential strategies for clinical treatment.

## Graphical abstract

The monkeypox virus (MPXV) has spread to many countries and caused thousands of infections all over the world. This review comprehensively introduces the biology, epidemiological characteristics of MPXV, and clinical manifestations of Mpox. The approaches for diagnosis, treatment, and prevention are also introduced, aiming to improve the general comprehension of Mpox.

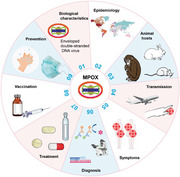

## 1 INTRODUCTION

Monkeypox virus (MPXV) is an enveloped double‐stranded DNA virus that can infect humans, rodents, and nonhuman primates.^[^ ^1^ ^]^ The incubation period following infection ranges from 6 to 13 days, with prodromal phase symptoms including fever, muscle aches, and swollen lymph nodes, followed by a rash that initially appears on the face and then spreads to other parts, including the genital area.^[^ ^1^, ^2^, ^3^ ^]^ MPXV belongs to the *Orthopoxvirus* genus of the *Poxviridae* family along with Variola (smallpox) virus, causing similar symptoms. However, it generally presents as a milder symptom compared to smallpox. Since the eradication of smallpox, MPXV has become the most concerned human orthopoxvirus. The mortality of Mpox ranges from 1% to 11%, and for some immunocompromised people, pregnant women, and infants, the rate can be higher.^[^ ^2^, ^4^ ^]^

Monkeypox, originally an endemic disease in Central and Western Africa, has predominantly originated from the Democratic Republic of Congo since its first identification in Zaire (now the Democratic Republic of Congo) in 1970, accounting for 95% of confirmed cases.^[^ ^5^ ^]^ While the outbreaks outside the endemic regions could be traced back to travelers and animals from Africa.^[^ ^3^, ^6^, ^7^ ^]^ The initial case of 2022 Mpox was reported in the United Kingdom on May 7, 2022, due to travel‐related transmission.^[^ ^8^ ^]^ The US CDC has reported a total of 97,934 confirmed cases with 184 deaths globally as of June 12, 2024 (https://www.cdc.gov/poxvirus/Mpox/response/2022/world‐map.html). Notably, this mortality rate is lower than that observed during previous epidemics. The outbreak of Mpox in 2022 exhibited distinct differences compared to previous outbreaks concerning susceptible populations, modes of transmission, clinical classification and symptomatology. Men who have sex with men (MSM), particularly those who did not practice safe sexual behavior were found to be primarily susceptible.^[^ ^9^ ^]^ It is worth noticing that patients did not exhibit typical clinical manifestations, such as fever or physical discomfort. However, genital skin lesions indicated a potential change in MPXV transmission mode.^[^ ^10^, ^11^ ^]^ To avoid racist stereotypes and stigmatizations, World Health Organization (WHO) recommended renaming the disease “Mpox” on November 28, 2022. Experts reached a consensus to designate the Central African strain as Clade I and the West African strain as Clade II, comprising two sub‐branches known as Clade IIa and Clade IIb (Monkeypox: experts give virus variants new names (who.int)). The 2022 MPXV exhibited the highest homology with Clade II strain, primarily associated with viruses carried by travelers from Nigeria in 2018 and 2019.^[^ ^12^ ^]^ In 2017, Nigeria experienced one of the largest national outbreaks of MPXV, involving 118 confirmed cases.^[^ ^13^ ^]^ Since then, several subsequent minor outbreaks have occurred in non‐endemic areas. However, these alerts did not arouse sufficient attention.^[^ ^14^ ^]^ Furthermore, since the beginning of the century, there has been a significant increase in both regional scope and number of cases.^[^ ^10^ ^]^ To make matters worse, the surveillance is ineffective in most areas, which facilitates its uncontrolled spread and exacerbates outbreak intensity. In recent months, there has been a surge in the incidence of clade I Mpox in Central African countries, particularly in the Democratic Republic of Congo (DRC). According to a report by the Centers for Disease Control and Prevention (CDC), individuals aged 15 years and younger account for 67% of suspected Mpox cases and 78% of suspected Mpox‐related deaths in the DRC. This indicates a different viral characteristic compared to Mpox cases reported in 2022 (https://www.cdc.gov/mmwr/volumes/73/wr/mm7319a3.htm).

Currently, there is no available vaccine or specific antiviral drug for Mpox. Brincidofovir and Tecovirimat, which have been approved for smallpox treatment, partly exhibited efficacy against MPXV.^[^ ^11^ ^]^ Although Tecovirimat failed to reduce the duration of Mpox lesions in a clinical trial launched by NIAID and INRB, the overall mortality among enrollees was much lower than the reported Mpox mortality (1.7% vs 3.6%) [https://www.nih.gov/news‐events/news‐releases/antiviral‐tecovirimat‐safe‐did‐not‐improve‐clade‐i‐mpox‐resolution‐democratic‐republic‐congo]. Randomized clinical trials are necessary to assess the efficacy of mentioned drugs irrespective of the authorization status. Moreover, JYNNEOS (also known as IMVAMUNE or Imvane), a smallpox vaccine approved for Mpox in 2019, offers some level of protection against MPXV but is not widely accessible.^[^ ^3^, ^15^ ^]^

In order to effectively control the global epidemic of MPXV, it is imperative to comprehensively understand its biology, epidemiological characteristics, and clinical manifestations, while concurrently developing enhanced approaches for diagnosis, treatment, and prevention. This review aims to consolidate existing knowledge and offer researchers a more refined perspective on vaccine design and disease control.

## 2 BIOLOGICAL CHARACTERISTICS OF MPXV

**FIGURE 1:**
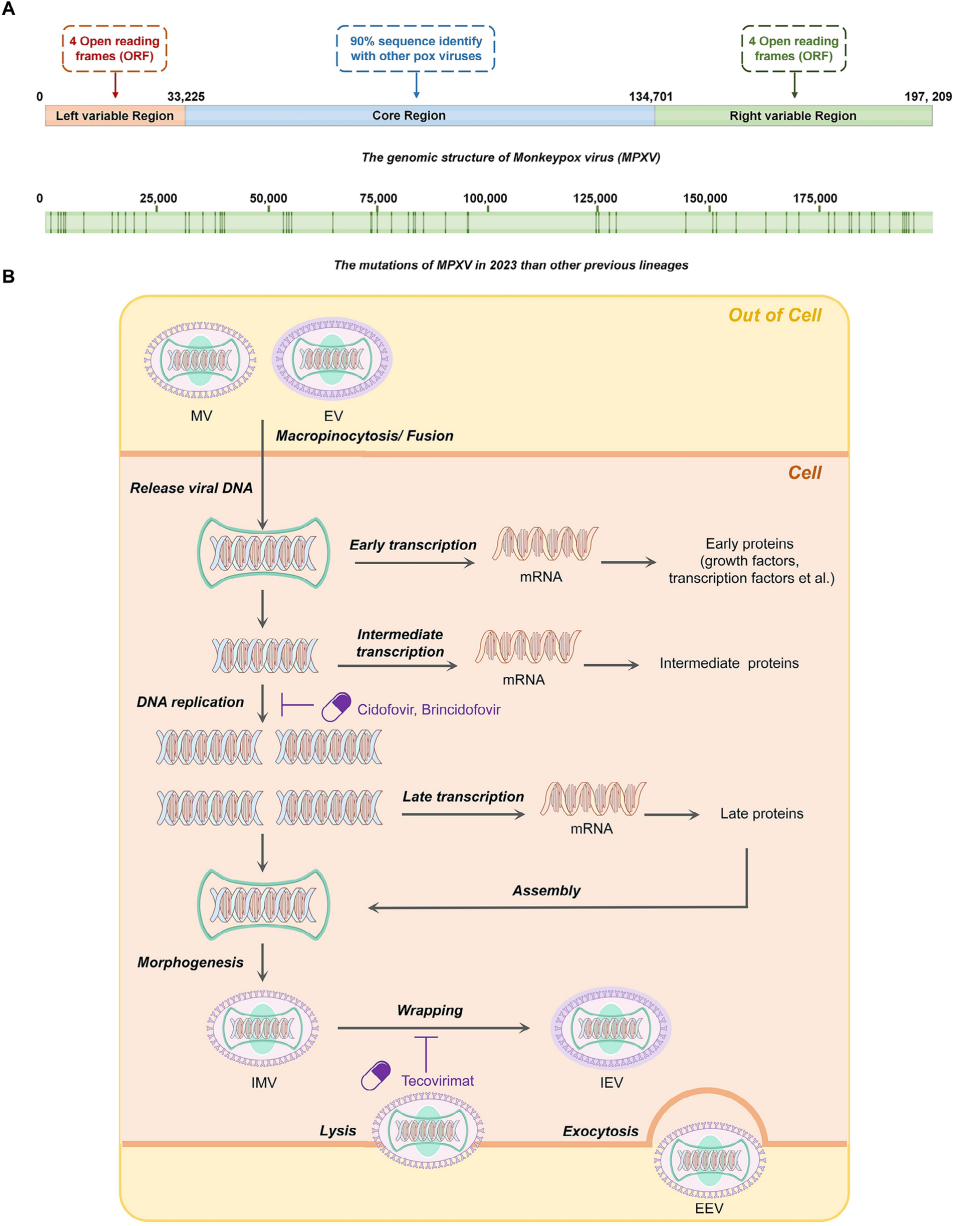
Biological characteristics of MPXV. (A) Genome structure of MPXV. The genome of MPXV is a large double‐stranded DNA structure of 197 kb length. The highly conserved central region is located at genomic nucleotide positions 56,000—120,000 and is flanked by variable ends that include inverted terminal repeats (ITR), comprising of a set of short tandem repeats and terminal hairpins. There are 190 non‐overlapping ORFs on the genome, four of which are located in reverse variable terminal repeat sequences. (B) Life cycle of MPXV. Mature virus (MV) attaches to the cell surface via interactions between viral ligands and supracellular receptors. And subsequently enters the cell either under neutral conditions via cell membrane fusion, or via an endosomal uptake pathway mediated by actin and a low pH, a pattern similar to the macrophage drinking mechanism. Enveloped viruses (EVs) remove the extra membrane envelope (becomes to MVs) prior to entry, and enter the cell in the same manner. After entering the host cytoplasm, the viral particles undergo three stages of gene expression, namely early, intermediate, and late stages. In the beginning, the virus initiates early transcription, and early proteins such as growth factors and transcription factors are expressed. Subsequently, DNA replication which is inhibited by Cidofovir and Brincidofovir happens, followed by intermediate/late transcription and expression. And the genomic DNA and late proteins are assembled to pre‐mature virion in the “cytoplasmic virus factory,” with morphogenesis changes, intracellular mature virus particles (IMVs) are formed. And IMV can be wrapped by the additional envelope to form intracellular enveloped virus particles (IEVs), specifically, the wrapping could be hindered by Tecovirimat. IEVs can be released from the cell by forming an actin tail to obtain extracellular enveloped virus particles (EEV), whereas IMV can only be released from the cell upon cell lysis. Currently, no treatment for MPXV infections has been approved. And United States Strategic National Stockpile (SNS) recommends clinicians to consider Tecovirimat, Brincidofovir, and Cidofovir (developed for use in patients with smallpox) as the options for the treatment of Mpox.

MPXV is a brick‐shaped, enveloped, and double‐stranded DNA virus belonging to the genus *Orthopoxvirus* (subfamily *Chordopoxvirinae*, family *Poxviridae*).^[^ ^16^, ^17^ ^]^ Although the central region of the MPXV genome encoding essential enzymes and structural proteins shares 96.3% identity with that of the smallpox virus, the terminal region responsible for virulence and host range factors exhibits distinct characteristics. This uniqueness indicates the independent evolution of MPXV as a separate species^[^ ^18^, ^19^ ^]^ (Figure 1A).

### 2.1 The morphology and structure of MPXV

The size of MPXV typically ranges from 200 to 250 nm, exhibiting an ovoid or brick‐shaped particle morphology and being encapsulated by a geometrically corrugated lipoprotein outer membrane.^[^ ^20^ ^]^ This membrane serves as a protective barrier for the dense core containing enzymes, a double‐stranded DNA genome, and transcription factors.^[^ ^14^, ^20^ ^]^ Similar to other orthopoxviruses, the viral core exhibits a complex polypeptide pattern necessary for viral decapsidation and genome replication, while specific polypeptides distinguishing MPXV from other orthopoxviruses are located in the surface and subsurface layers.^[^ ^21^, ^22^ ^]^ Electron microscopy fixation artifact reveals that the core appears biconcave with lateral bodies on each side.^[^ ^20^ ^]^

Poxvirus‐infected cells produce two distinct types of mature infectious MPXV particles: intracellular mature virus (IMV) and extracellular enveloped virus (EEV), the latter possessing an additional membrane envelope.^[^ ^23^ ^]^

### 2.2 MPXV genomics

#### 2.2.1 Evolutionary branches of MPXV

There are two phylogenetic branches of MPXV, namely Clade I and Clade II. Notably, Clade I demonstrates higher virulence compared to Clade II,^[^ ^24^, ^25^ ^]^ and is characterized by unique attributes. First, it possesses a gene encoding an MPXV complement enzyme inhibitor, which serves as a crucial immunomodulatory factor absent in Clade II. Second, infection with Clade I strain may lead to selective silencing of host immune‐related gene transcription. Third, virulence factors associated with Clade I selectively dampen host cell responses including cell growth and proliferation, apoptosis, and immune surveillance.^[^ ^25^, ^26^, ^27^, ^28^, ^29^ ^]^

#### 2.2.2 Genome structure of MPXV

The genome of MPXV is a large double‐stranded, approximately 197 kb in length with over 200,000 base pairs, and viral size is about seven times longer than that of SARS‐CoV‐2 and > 20 times longer than that of HIV^[^ ^12^, ^14^ ^]^ (Figure 1A). The central highly conserved region, spanning nucleotide positions 56,000 to 120,000, encodes essential enzymes and structural proteins, and it is flanked by variable ends containing inverted terminal repeats (ITRs).^[^ ^14^, ^30^ ^]^ The ITRs includes a set of short tandem repeats and terminal hairpins.^[^ ^31^ ^]^ In the genome, 190 non‐overlapping open reading frames (ORFs) are greater than 180 nt in length, 4 of which are located in reverse variable terminal repeats.^[^ ^14^, ^32^ ^]^

MPXV genome harbors major orthopoxvirus genes in its highly conserved central region while specific differences between MPXV and other orthopoxviruses primarily located in the terminal ends.^[^ ^20^, ^31^ ^]^ Previous studies have demonstrated that the vaccinia virus complement control protein (VCP), an encoded regulator of complement activation secreted from infected cells, contains a total of 4 short consensus sequences (SCRs).^[^ ^33^ ^]^ The premature termination of the VCP ORF of MPXV results in a truncated protein sequence lacking the C‐terminal SCR4, which represents an essential distinction between MPXV and other orthopoxviruses.^[^ ^33^, ^34^ ^]^

Understanding viral genomics enables a comprehensive analysis of the MPXV infection process and facilitates the identification of relevant therapeutic targets. Targeting the central region is of great importance in the treatment of orthopoxvirus genera. Studies utilized CRISPR‐AAV particles to deliver CRISPR components, including three essential genes (A17L, E3L, and I2L) conserved within this genus. Deletion of these genes not only impacts viral production in vitro but also diminishes cytopathic effects (CPE) in host cells.^[^ ^35^ ^]^

### 2.3 The life cycle of MPXV

MPXV is a DNA virus, similar to other orthopoxviruses, and the complete life cycle occurs in the cytoplasm^[^ ^20^ ^]^ (Figure 1B). Therefore, all proteins required for viral replication, transcription, particle assembly, and exocytosis must be encoded by the MPXV genome.^[^ ^20^, ^36^ ^]^ Due to the remarkable nucleotide and protein homology as well as a similar life cycle of orthopoxviruses, VACV is often used as a model for more dangerous orthopoxviruses, and much of our understanding of orthopoxviruses has been derived from studies on VACV.^[^ ^23^, ^35^, ^37^ ^]^

Briefly, when orthopoxvirus particles attach to the cell, they enter the cells by membrane fusion or endocytosis. Inside the cells, enzymes necessary for genome replication and transcription, within the highly conserved central region, are synthesized. Subsequently follows a series of events including replication, transcription translation, and assembly of IMV and EEV particles.

#### 2.3.1 Cell entry of MPXV

Mature viruses attach to the cell surface through interactions between viral ligands and supracellular receptors. They enter the cell either via neutral conditions by fusing with the cell membrane or through an endosomal uptake pathway involving actin and low pH, resembling macropinocytosis.^[^ ^38^ ^]^

The O3 protein, a conserved component of the entry fusion complex (EFC) in most orthopoxviruses, exhibits two or three amino acid substitutions in MPXV.^[^ ^39^ ^]^ In VACV, 9 of 12 highly conserved non‐glycosylated transmembrane proteins are known constituents of the EFC.^[^ ^23^ ^]^ Each component protein plays a crucial role in the membrane fusion of the virus, and the absence of any one destabilizes the entire complex, allowing viral adsorption but preventing cellular entry.^[^ ^39^ ^]^

MPXV is believed to bind to various cell receptors via its EFC consisting of multiple proteins. For example, VACV can activate epidermal growth factor receptor (EGFR), triggering a signaling cascade that induces significant changes in actin dynamics and subsequent large‐scale internalization of viral particles into HeLa cells.^[^ ^40^ ^]^ In this study, Iressa and 324674 (Calbiochem) were used as inhibitors of EGFR to demonstrate that EGFR is required for viral entry into HeLa cells but not for subsequent steps. Furthermore, CHO cells lacking EGFR were infected by the virus through an alternative pathway unrelated to macrophagocytosis.^[^ ^40^ ^]^ In addition, Eppstein et al. suggested that the vaccinia virus utilizes the EGFR as an entry receptor to infect L cells, and antibodies against EGFR reduce viral infections.^[^ ^41^ ^]^ Although limited studies have been conducted on cellular receptors for MPXV, data from VACV imply that diverse macropinocytosis pathways utilized by MPXV may also be influenced by changes in actin dynamics resulting from relevant activation.

Previous studies demonstrated the essential role of mature virus (MV) entry proteins in the transmission of enveloped vaccinia virus (VACV). Consequently, prior to fusion, it is necessary to remove the additional membrane envelope. However, this membrane is prone to fragmentation either before or during infection.^[^ ^23^ ^]^ Following this, the exposed mature viral particles fuse with either the plasma membrane or endocytic vesicles for cellular transport.^[^ ^23^ ^]^

By employing RNA Sequencing and pooled short hairpin RNA (shRNA) screening techniques, Filone et al.^[^ ^42^ ^]^ demonstrated that HSF1 is a plausible host factor supporting orthopoxvirus infection, and its activation facilitates viral replication. The Conserved Oligomeric Golgi (COG) complex comprises two lobes containing eight heterodimeric subunits (lobes A and B consist of subunits COG1−4 and COG5−8, respectively), functioning within the Golgi apparatus.^[^ ^43^ ^]^ Through haploid genetic screening methods, Realegeno et al.^[^ ^44^ ^]^ initially identified COG3, COG4, COG7, and COG8 as possible host factors associated with MPXV infection. Subsequently, knockout experiments confirmed an important role for the COG complex in orthopoxvirus infection as its absence impaired crucial virus‐cell interactions required for both entry and exit.^[^ ^43^ ^]^

#### 2.3.2 Replication, transcription, and translation of MPXV

MPXV exhibits unique replicative properties of poxviruses, as it is restricted to the cytoplasm of infected cells and functions independently from the host nucleus.^[^ ^45^ ^]^ The gene expression process of MPXV can be categorized into early, intermediate, and late stages.^[^ ^38^ ^]^ Once the virus enters the cytoplasm, it releases pre‐packaged proteins and enzymatic factors that disable the cellular defense mechanisms and stimulate early gene expression.^[^ ^38^ ^]^ Early protein synthesis promotes the degradation of proteosome, DNA replication, and production of intermediate transcription factors.^[^ ^38^ ^]^ Orthopoxviruses, including VACV and MPXV, encode ribonucleotide reductase (RR) proteins comprising R1 and R2 subunits.^[^ ^46^ ^]^ During viral replication, the R2 subunit forms a functional complex with the R1 subunit, ensuring sufficient deoxynucleoside triphosphate (dNTPs) availability for replication purposes.^[^ ^46^ ^]^

Following viral DNA replication and the production of intermediate transcription factors, intermediate genes are transcribed and translated to induce the expression of late genes, that primarily function as structural proteins, enzymes, and early transcription factors.^[^ ^38^ ^]^

#### 2.3.3 Maturation and release of MPXV

Extensive studies on the mechanism of VACV infection have shown that following IMV formation, the virus undergoes packaging with additional membranes to form intracellular enveloped virus (IEV) particles, which are transported toward the periphery of the host cell via a kinesin/microtubule transport system.^[^ ^47^ ^]^ Subsequently, IEVs fuse with the plasma membrane of the host cell to form cell‐associated enveloped virus (CEV) particles, shedding one of their two outer membranes.^[^ ^47^ ^]^ CEVs can trigger actin polymerization, which pushes particles of actin‐filled membrane protrusions (‘tails’) into adjacent cells and can detach from the tail or directly from the membrane to form EEVs.^[^ ^47^ ^]^ The process involved in poxvirus actin tail formation and EEV release is highly conserved among poxviruses, and MPXV is expected to generate an actin tail and export EEV from cells before cell death in a similar way.^[^ ^47^, ^48^ ^]^ It is important to note that if IMVs do not undergo coating by additional membranes to become IEVs, then particle release only occur during the cell lysis.^[^ ^43^ ^]^

### 2.4 MPXV and host immunity

#### 2.4.1 MPXV's resistance to host immune defense

Viral replication and proliferation usually require multiple mechanisms to counteract the antiviral responses of the host immune system.^[^ ^49^, ^50^ ^]^

Among MPXV proteins, there is an important class of complement‐binding proteins, including D14L and D15L.^[^ ^49^, ^50^ ^]^ The complement immune response plays an essential role in defending against invading pathogens and can ultimately lead to viral inactivation through neutralization, modulation, viral particle cleavage, or phagocytosis.^[^ ^49^ ^]^ Complement‐binding proteins of the MPXV bind with complement components to obstruct the complement cascade, enabling the virus to evade the complement‐mediated assault and enhance its virulence.^[^ ^34^ ^]^

VACV inhibits apoptosis via the expression of cytokine response modulator A (Crm A). It also hinders apoptosis induced by RNA‐dependent protein kinase (PKR) through specific PKR inhibitors encoded by the E3L and K3L genes. Additionally, it prevents apoptosis triggered by loss of outer mitochondrial membrane potential via F1L gene expression.^[^ ^38^, ^51^, ^52^ ^]^ Although limited information is available regarding apoptosis activity in cells infected with MPXV, studies have demonstrated that MPXV encodes a direct homolog of the VACV F1L gene that acts similarly to Bcl‐2 on mitochondria.^[^ ^38^ ^]^ Additionally, MPXV exhibits reduced dsRNA accumulation compared to the wild‐type VACV during infection, potentially impeding the activation of host antiviral PKR dependent on dsRNA and thereby facilitating viral infection.^[^ ^53^ ^]^ Ankyrin protein (ANK) repeat encoded by MPXV genome can directly interact with NF‐κB1/p105 leading to the inhibition of NF‐κB signaling pathway.^[^ ^54^ ^]^ Rubins et al.^[^ ^26^ ^]^ proposed that MPXV selectively represses the expression of genes responsible for activating cellular signaling pathways involved in the innate immune response, such as TNF‐α, IL‐1α and IL‐1β, CCL5, and IL‐6.

### 2.5 Host immune defense against MPXV

**FIGURE 2:**
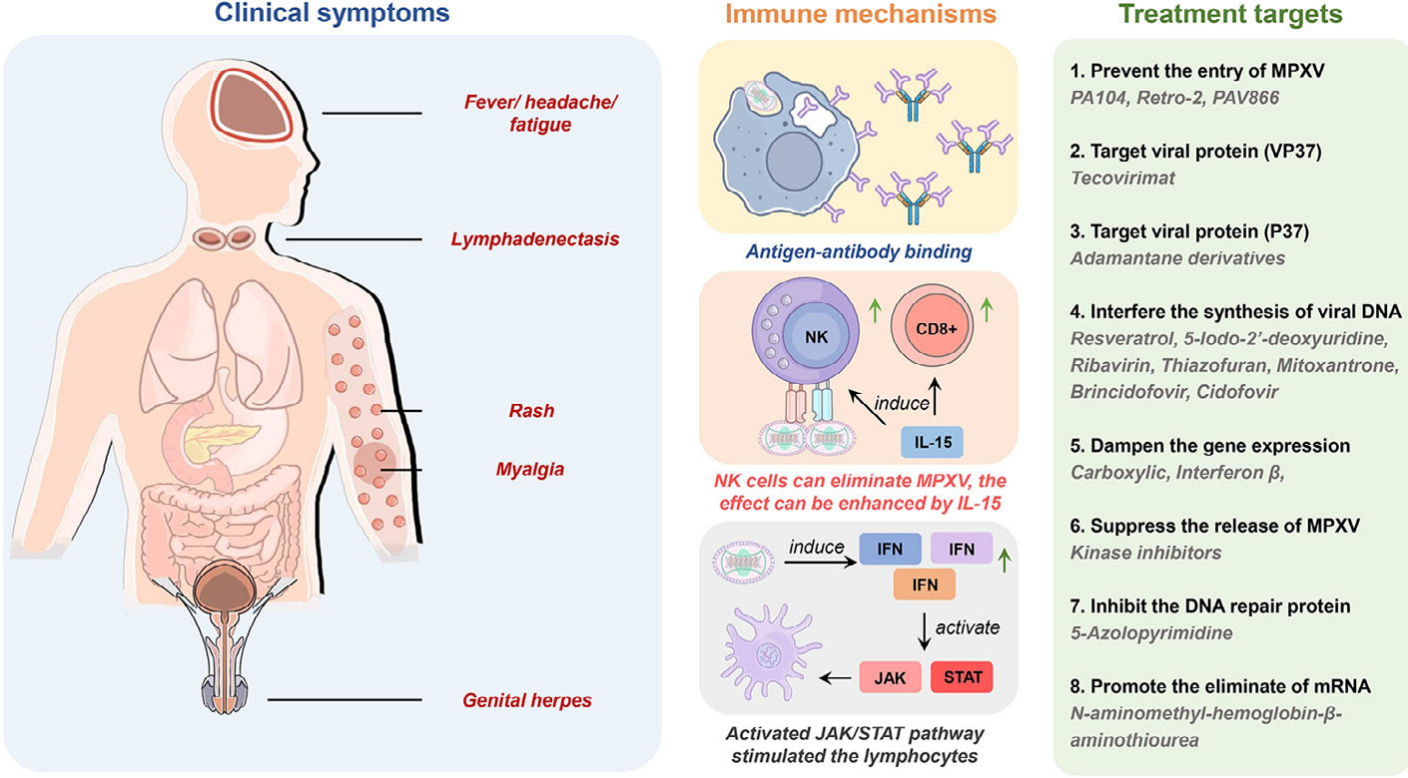
Symptoms of Mpox, immuno‐mechanisms, and treatment targets of MPXV. After the incubation period of MPXV, except for skin rashes on the head, face, trunk, limbs, nail bed, anus, and genitals, patients generally have symptoms such as fever, cough, sore throat, fatigue, muscle pain, headache, swollen lymph nodes, etc. A few patients experience symptoms such as nausea, vomiting, and diarrhea due to proctitis. Typical features of systemic viral invasion include swollen lymph nodes in the neck, armpits, and groins. Three types of immune defense mechanisms are involved in MPXV infection, namely antigen‐antibody binding (e.g., JYNNEOS vaccine), host interferon response, and interferon‐mediated signaling pathway. IL‐15 therapy can induce the production of NK and CD8^+^ cells, and increased NK cell counts prevent fatal MPXV infection. MPXV activates IFN‐1 secretion in almost all cell types, and IFN‐1 activates the JAK/STAT pathway in an antiviral state. The drug targets of MPXV are summarized on the right side. The targets of MPXV inhibition and related drugs were as followed.

The immune defense against MPXV infection involves three distinct mechanisms, namely antigen‐antibody binding (e.g. JYNNEOS vaccine), host interferon response, and interferon‐mediated signaling pathway. MPXV triggers IFN‐1 secretion in various cell types, which subsequently activates the JAK/STAT pathway to establish an antiviral state. IL‐15 therapy can induce the production of NK and CD8+ cells, and increased NK cells play a crucial role in preventing fatal MPXV infection. However, the specific contributions of CD8+ and CD4+ cells to the defense against MPXV infection remain unclear^[^ ^55^ ^]^ (Figure 2).

## 3 EPIDEMIOLOGY OF MPXV

MPXV was initially separated in laboratory‐housed *Macaca fascicularis* in 1958 in Copenhagen, Denmark. In 1970, the first case of MPXV infection in humans was recorded in the Democratic Republic of the Congo (DRC).^[^ ^56^ ^]^ Mpox primarily affected Central and West African countries, with sporadic outbreaks occurring due to international travel or animal imports in Israel, Singapore, the UK, and the USA.^[^ ^57^, ^58^ ^]^ Some cases may result from human‐to‐human transmission. In 2018, three cases of MPXV infections were reported in the UK originating from a healthcare worker returning from Nigeria.

The 2003 US MPXV outbreak marked the first instance of MPXV infection outside Africa.^[^ ^59^ ^]^ Prior to 2022, most MPXV infections were reported from DRC and Nigeria. However, since May 2022, several countries including the USA and the UK have reported cases without direct epidemiological links to West or Central Africa.

**FIGURE 3:**
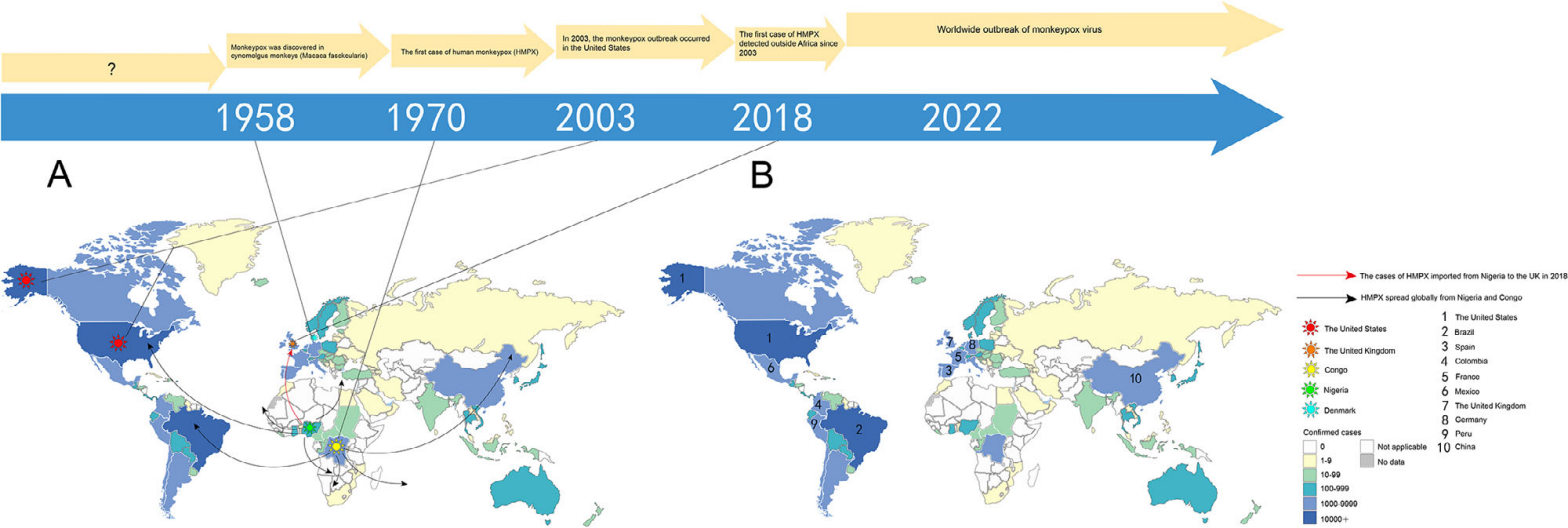
Global epidemic distribution of human Mpox cases from 1958 to September 30, 2023. (A) The timeline of monkeypox infection. (B) The top ten countries with confirmed cases of human Mpox in the world are the USA (*n* = 30,636), Brazil (*n* = 10,967), Spain (*n* = 7611), France (*n* = 4158), Colombia (*n* = 4090), Mexico (*n* = 4062), Peru (*n* = 3812), the UK (*n* = 3805), Germany (*n* = 3708), and China (*n* = 1794). Together, these countries account for 81.9% of reported cases globally (the retrieval date is the public data from the WHO, published on October 20, 2023).^[^ ^244^ ^].^

As of June 12th, 2024, there have been a total of 97,281 laboratory‐confirmed cases and 184 deaths reported (https://www.cdc.gov/poxvirus/monkeypox/response/2022/world‐map.html). Further research is necessary to investigate transmission modes as well as prevalence and incidence rates for a better understanding of Mpox's epidemiology and effective management during future outbreaks (Figure 3). It is worth noting that the cases of clade I Mpox are increasing in Central African countries, particularly in the DRC, and WHO has declared the highest level of alert for Mpox. The surveillance of MPXV is essential for the prevention and control of the epidemic, particularly in light of the current coexistence of the two strains.

### 3.1 Co‐infection of MPXV with other viruses

Co‐infection and occupational exposure are epidemiological concerns. In 2003, an outbreak of MPXV infection associated with occupational exposure occurred in all 19 cases involving prairie dogs in Wisconsin.^[^ ^60^ ^]^ Between 2009 and 2014, 134 (12.1%) of the 1107 suspected cases in the DRC were confirmed as MPXV/Varicella zoster virus (VZV) co‐infections.^[^ ^61^ ^]^ In May 2022, an HIV‐positive patient receiving antiretroviral therapy was confirmed with MPXV infection in Australia.^[^ ^62^ ^]^ Co‐infection with HIV is generally associated with more severe symptoms. Currently, individuals infected with MPXV exhibit corresponding symptoms, making them easier to be identified.^[^ ^63^ ^]^

### 3.2 Prevalence and incidence of Mpox

Prevalence and incidence studies are crucial for understanding Mpox epidemiology. Epidemiological studies offer valuable insights into the distribution, etiology, and treatment of Mpox.

In the 1980s, a hemagglutination inhibition test was used to screen approximately 3460 people in the Zaire area, the total infection rate was about 19% (667 people). MPXV‐specific antibodies were detected in the serum of 27 individuals with an overall prevalence of 0.8%. No significant difference in prevalence was observed between males and females. In addition, children aged 0−4 and 5−9 years exhibited prevalence rates of 0.3% and 1.3%, respectively, while children aged 15−19 years showed rates up to 2.4%.^[^ ^64^ ^]^ In 2016, 26 Mpox cases were diagnosed in the Central African Republic, with an overall incidence of 0.5% (5 per 1000 inhabitants) and a case fatality rate of 7.7%. Among these cases, severe clinical symptoms were manifested in 87.5% of patients, predominantly children and individuals unvaccinated against smallpox.^[^ ^65^ ^]^ Nigeria reported 122 MPXV infections in 2017, resulting in a mortality rate of 6% (seven cases).^[^ ^66^ ^]^

The recent outbreak of Mpox in Europe is attributed to the Clade II, which exhibits a mortality rate of 3.6%, with higher rates observed among young children.^[^ ^67^ ^]^ Clade I induces mortality rates as high as 10%, whereas Clade II causes about 1% of death cases. It is believed that Clade II is responsible for the 2022 outbreak.^[^ ^68^ ^]^

The basic reproductive number (*R*_0_) and the particle‐to‐plaque ratio (P:PFU) are usually used to measure the virologic infectivity. When *R*_0_ > 1, it indicates that the virus has the potential to spread within a population, whereas if *R*_0_ < 1, it lacks this possibility. The P:PFU measures the proportion of viral particles capable of infecting susceptible cells in tissue culture under idealized in vitro conditions. When P:PFU approaches 1, as occurs with bacteriophages, each viral particle is able to complete an infectious cycle in a susceptible cell (i.e. highly infectious to the cells in tissue culture). For smallpox, P:PFU reaches 1−100, and *R*_0_ reaches 6.87. The related data of MPXV is unknown. These findings suggest that poxviruses possess significant potential for spreading.^[^ ^69^ ^]^

## 4 ANIMAL HOSTS

**FIGURE 4:**
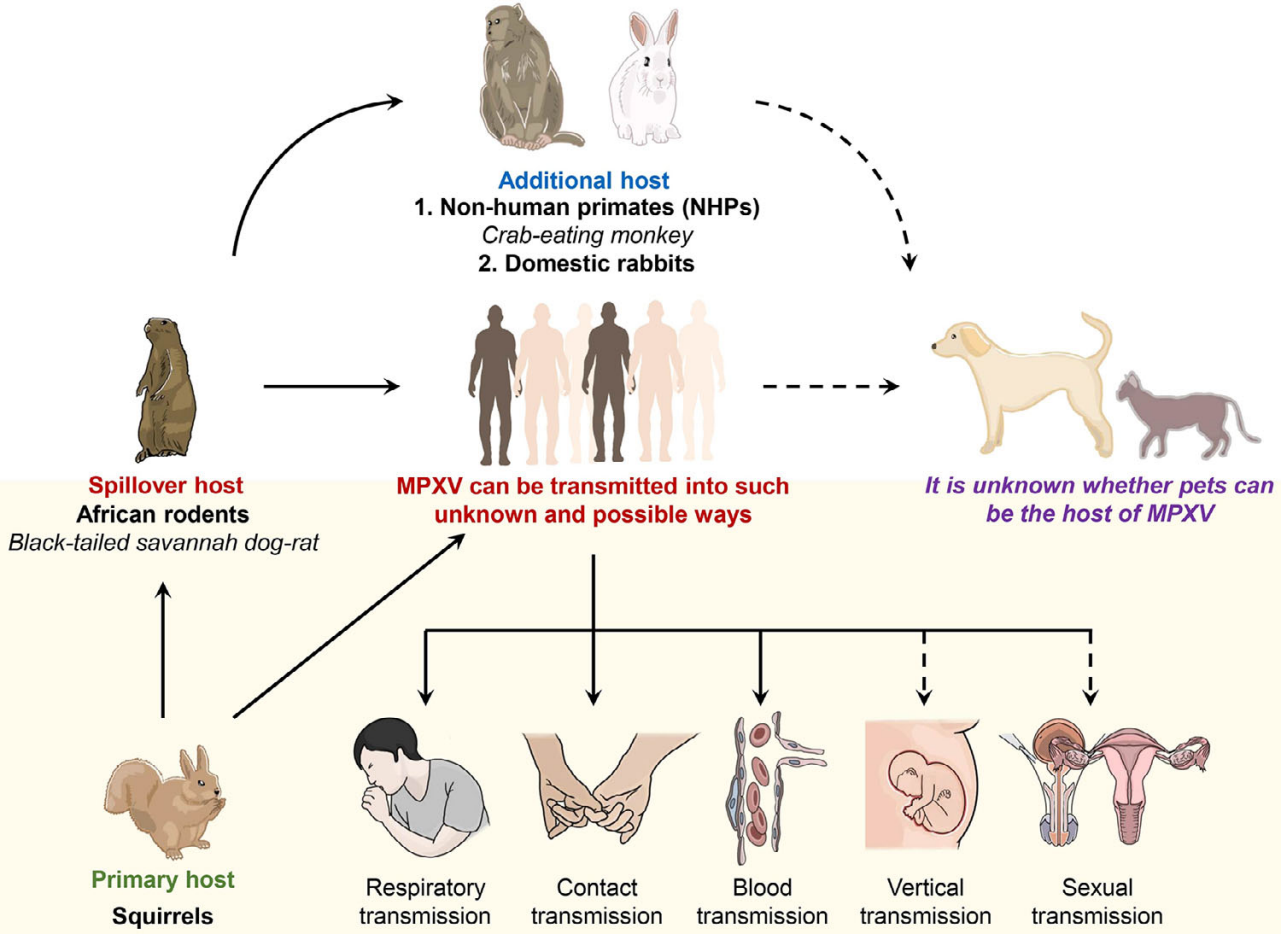
The hosts and transmission of MPXV. It is generally accepted that squirrels are the primary hosts of MPXV. Black‐tailed prairie dogs infected with MPXV spilled out of Africa and spread the virus to humans and other animals. Rabbits, monkeys, orangutans, etc., have become animal hosts of MPXV by accident, and until now, it has been difficult to prove that the transmission of MPXV is clearly related to non‐human primates. MPXV can be transmitted through direct contact, respiration, and blood, and increasing evidence suggests that patients can be infected through sexual contact and vertical transmission.

Studies on the hosts of MPXV initially focused on monkeys, considering them as accidental hosts. However, subsequent research suggests that rodents may serve as the natural hosts for MPXV (Figure 4). It is generally accepted that squirrels are the primary reservoirs for MPXV. Additionally, the transmission of MPXV to humans and other animals, such as rabbits, monkeys, orangutans, etc., occurred when Black‐tailed prairie dogs infected with MPXV were introduced from Africa. The 2003 outbreak of MPXV infection in the United States was attributed to the pet trade and subsequent human bites by prairie dogs.^[^ ^70^ ^]^ Traceback investigations identified the MPXV cases on at least one Gambian giant rat, two rope squirrels, and three dormice during a 2003 international shipment.^[^ ^19^ ^]^

Previous studies indicate that rodents act as primary vectors for MPXV transmission. Moreover, all known poxvirus ancestors have spread from rodents to other mammalian host.^[^ ^71^ ^]^ Animals such as monkeys and anteaters are regarded as incidental hosts,^[^ ^72^ ^]^ while it remains unclear whether non‐human primates play a role in MPXV transmission.

### 4.1 Animal models

To study MPXV, it is essential to use appropriate animal models to simulate natural transmission at similar viral doses and to generate comparable rates of pathogenesis, morbidity, and mortality.^[^ ^73^ ^]^ Animal models, particularly natural hosts, are crucial for evaluating the efficacy of antiviral drugs and vaccines.^[^ ^74^ ^]^

Several animal models for MPXV including non‐human primate models such as crab‐eating macaques and rhesus monkeys, rodent models such as *Graphiurus kelleni* and *Cynomys ludovicianus*, and transgenic animal models such as STAT1‐knockout mice, J(H) knockout mice,^[^ ^75^ ^]^ and inbred mice (CAST/EiJ) have been established.^[^ ^76^ ^]^ These animal models typically exhibit reduced activity, increased weight loss, lung inflammation, and bronchiectasis due to fibrotic necrosis of the lungs. Viral replication occurs in the lungs, spleen, lymph nodes, nasal mucosa, and abdominal organs. Cidofovir, Tecovirimat, resveratrol, ribavirin, neutralizing antibodies, and small interfering RNA (siRNA) displayed effective anti‐MPXV activities in vitro and in vivo.^[^ ^77^ ^]^ Furthermore, inactivated DNA and subunit recombinant vaccines exhibited effective antiviral activities in animal models.^[^ ^77^ ^]^

#### 4.1.1 Common non‐human primate models

In crab‐eating macaques exposed to aerosolized MPXV, viral DNA was detected in the lungs two days post‐infection and viral antigens were detected in the bronchial epithelium, bronchioles, and alveolar walls through immunostaining. Developmental abnormalities and exfoliative cell lesions were rarely observed in the fine bronchi of the respiratory tract. By day 4, viral DNA was detected in the pharynx, tonsils, and spleen, meanwhile, viral antigens were detected in the lungs, submandibular lymph nodes, spleen, and colon. Lung lesions progressed to localized epithelial necrosis and inflammation. The maximum body temperature was reached on day 6, and pox‐like lesions began to appear on the skin, with confirmed lesions observed in the lungs, tonsils, spleen, lymph nodes, and colon. On day 8, viral DNA was detected in 70% of tissues. Blood levels of interleukin 1ra (IL‐1ra), IL‐6, and gamma‐interferon (IFN‐γ) were significantly increased. By day 10, circulating IgG antibody concentrations started to increase, and by day 12, the animals showed early signs of recovery before returning to full health. These results demonstrate several similarities between crab‐eating macaques and humans regarding early MPXV infection, making them a suitable alternative model.^[^ ^78^ ^]^ Typical poxvirus symptoms can be observed in crab‐eating macaques, and the bovine pox virus detected in the bronchi shares many features with both MPXV and smallpox viruses.^[^ ^79^ ^]^

To develop a novel vaccine, scientists conducted a comparative study between the highly attenuated modified poxvirus vaccine Ankara (MVA) and the licensed Dryvax vaccine using a standardized monkey model infected with MPXV. The experimental group received either two doses of MVA or a combination of MVA and Dryvax inoculation. After the virus challenge, it was observed that only a small number of skin lesions appeared in the group receiving the combination of MVA+Dryvax.^[^ ^80^ ^]^

In 2012, researchers established an inhalation model of MPXV infection in crab‐eating macaques by aerosolizing MPXV through impaction nebulization using liquid and gelatin filters, effectively infecting the alveoli. The duration of the disease and the lethal dose obtained after inhalation in monkeys by head exposure were similar to previously published data.^[^ ^81^ ^]^

In 2013, researchers used a crab‐eating macaque model to evaluate two vaccines against the disease (ACAM2000 and IMVAMUNE), and found that animals receiving a single dose of IMVAMUNE were not completely protected from infection, whereas those receiving a single booster dose of IMVAMUNE or ACAM2000 completely evaded infection.^[^ ^80^ ^]^

Marmoset is another popular model owing to their susceptibility to MPXV,^[^ ^82^ ^]^ and the similarity in the pathological features with human infection, such as high viremia.

#### 4.1.2 Rodent models

All ground squirrels infected with MPXV through intraperitoneal and intranasal inoculation developed Mpox symptoms and died within 6–9 days after infection. The pathological features of MPXV infection in ground squirrels were similar to those in primates, suggesting that ground squirrels can serve as appropriate models for drug studies.^[^ ^83^ ^]^

The ectromelia virus (ECTV) is widely recognized as an alternative to MPXV in mouse models due to its high infectivity at extremely low titers and close relationship with MPXV.^[^ ^84^ ^]^ Yang et al. have reported the efficacy of Tecovirimat in normal, immunocompetent, and immunodeficient BALB/c mice.^[^ ^85^ ^]^

Additionally, prairie dogs,^[^ ^74^ ^]^ dry otters,^[^ ^86^ ^]^ and domestic rabbits^[^ ^87^ ^]^ are commonly used transgenic models that have significantly contributed to research efforts aimed at preventing and treating infections caused by MPXV.

## 5 TRANSMISSION CHARACTERISTICS OF MPXV

Human infections are typically associated with close contact with an infected animal or person. Humans can be infected by animals, such as rodents and primates. Squirrels, in particular, are considered important intermediate hosts and possible sources.^[^ ^88^ ^]^ Common modes of transmission include direct contact with the diseased skin or mucous membranes of cases, as well as contact with virus‐contaminated objects, prolonged close inhalation of pathological respiratory droplets, contact with respiratory secretions and diseased exudates, blood and other body fluids of infected animals, or infections from bites or scratches of infected animals^[^ ^89^ ^]^ (Figure 4). Children, pregnant women, immunocompromised individuals, and rural indigenous populations that rely on wildlife for survival face a higher risk of infection. The risk of infection and symptoms is related to the duration and intensity of exposure.^[^ ^90^ ^]^

According to the WHO, vertical transmission of MPXV from mother to fetus may occur through the placenta or close contact during or after birth.^[^ ^91^ ^]^ There is no evidence that the virus can be transmitted through breastfeeding or direct contact with the mother's infected skin.^[^ ^91^ ^]^

Since 2022, several outbreaks of Mpox in non‐endemic countries have highlighted the atypical nature of the disease, and the rapid surge in Mpox cases between May and June 2022 has instigated public alarm. Recent studies of Mpox outbreaks across multiple nations have revealed a significant prevalence among MSM. A study conducted by researchers at the UK Health and Safety Agency (UKHSA) demonstrated that 151 of 152 Mpox cases were attributed to MSM,^[^ ^92^ ^]^ aligning with similar outbreak patterns observed through epidemiological surveys in other countries.^[^ ^11^ ^]^

Among the recently diagnosed 54 Mpox cases in the UK, 13 (24%) were HIV‐positive, while 5 (9%) exhibited more severe symptoms necessitating hospitalization, and those with no epidemiological link to endemic areas were identified as MSM.^[^ ^9^ ^]^ In June 2022, 521 Mpox cases reported in Germany were male, and 69% of cases in Berlin were MSM.^[^ ^93^ ^]^ Similarly, all 277 confirmed Mpox cases in France during the same period were males, with a large proportion being MSM (not all cases), and no travel history to endemic areas was reported.^[^ ^94^ ^]^ Among the 48 patients diagnosed in Madrid between May and June 2022, all were male, with MSM accounting for 87.5%.^[^ ^95^ ^]^ As of 22 June, Madrid had recorded a total of 508 male Mpox cases out of which approximately 99% occurred among males. An epidemiological survey revealed that 84.1% of patients had engaged in sexual intercourse within the three weeks prior to the onset of symptoms, and 93% of them were MSM (*n* = 397).^[^ ^96^ ^]^ Persistent Mpox outbreaks in 2022 with an *R*_0_ (basic number of infections) > 1, resulted in expanding outbreaks to young and unvaccinated populations.^[^ ^15^ ^]^ Multi‐national outbreaks are mostly observed in, but not limited to, MSM.^[^ ^97^ ^]^ Transmission of Mpox primarily occurs through intimate contact, and skin‐to‐skin contact during sexual intercourse may be a major factor in transmission.

In addition to the well‐known rash, diarrhea occurs in 22% of infected individuals during the early stages of infection (https://www.who.int/news‐room/fact‐sheets/detail/Mpox). Important questions that need to be addressed include whether diarrhea is the initial symptom and if there are key receptors for MPXV in intestinal cells.^[^ ^98^, ^99^ ^]^ Prolonged shedding of MPXV DNA in the upper respiratory tract was detected after the rash subsided. In addition, MPXV forms reservoirs in the urogenital tract.^[^ ^6^, ^11^, ^100^ ^]^ Aerosols from infected individuals carry MPXV, which can survive in suspension for 90 h. Nevertheless, the risk of infection by aerosolized virus particles is considered to be low in most cases.^[^ ^68^, ^101^, ^102^ ^]^

In general, the occurrence of Mpox and transmission speed of MPXV depend on social, economic, and environmental factors, rather than purely biological factors.^[^ ^103^ ^]^ From an ecological perspective, the increase in Mpox incidence may also be related to the eradication of the variola virus, which created a vacant ecological niche.^[^ ^104^ ^]^ An ecological niche model (ENM) that combines remote sensing and environmental data has shown that changes in precipitation can promote the spread of MPXV from animals to humans.^[^ ^105^, ^106^ ^]^ This ENM approach can predict the spatial distribution of MPXV and potentially identify high‐risk animal vectors.^[^ ^107^ ^]^

## 6 CLINICAL SIGNS AND SYMPTOMS

The MPXV is reported to possess a large, flexible genome that allows for significant structural changes, resulting in gene loss or gain, and accelerating alterations in the viral phenotype.^[^ ^108^ ^]^ It is worth noting that there are certain clinical differences between the current epidemic strain and previous ones. Previously, aside from transmission to other animals, the primary mode of infection was through disease transmission from African wildlife species to humans.^[^ ^109^ ^]^ However, unlike zoonosis in past outbreaks, recent virus transmission occurs primarily through person‐to‐person contact with male homosexual or bisexual sex being the most suspected mode.^[^ ^110^ ^]^ Transmission between people without close contact is considered unlikely or very low under present circumstances.^[^ ^111^ ^]^ In addition, new clinical symptoms such as rectal pain and penile swelling (edema) have emerged compared to previous outbreaks. Simultaneously, predominantly localized lesions, instead of extensive disseminated lesions, result in lower concentrations of viremia and less virus presence in respiratory excretions. Consequently, the respiratory route becomes less significant while direct contact via dermal inoculation continues to perpetuate both clinical presentation and transmission cycles.^[^ ^112^ ^]^

Clinical signs of the MPXV infection are evident both externally and internally throughout the body (Figure 2). Generally speaking, Mpox has a long clinical course, and the prodromal symptoms are mild and difficult to detect.^[^ ^113^ ^]^ The cycle of infection can be divided into an incubation phase, a prodromal phase, and the appearance of a rash (which may resemble syphilis or chickenpox and may lead to confusion in diagnosis). This rash can further be divided into macular, papular, blistering, pustular, and crusting phases, before disappearing along with other symptoms.^[^ ^114^ ^]^

The clinical presentation of Mpox is very similar to that of the common form of smallpox, with an incubation period of 3−20 days (average 7 days).^[^ ^115^ ^]^ Patients commonly experience fever (62%), cough (41%), sore throat (63%), fatigue (41%), muscle pain, headaches (27%), and swollen lymph nodes (56%).^[^ ^3^, ^116^ ^]^ Some patients also experience diarrhea,^[^ ^99^ ^]^ nausea, and proctitis directly caused by the virus.^[^ ^113^ ^]^ Swollen lymph nodes in the neck, axilla, and groin are typical features during the initial stages of viral invasion,^[^ ^3^ ^]^ which help distinguish Mpox from smallpox.^[^ ^68^ ^]^ However, in atypical cases without pathognomonic skin symptoms, there is no effective test for distinguishing Mpox and smallpox.^[^ ^117^ ^]^ Healthcare professionals should prioritize patients with unexplained diarrhea.^[^ ^98^ ^]^ Within 1–3 days after fever onset, it is accompanied by a rash (usually 3−15 mm in diameter) that spreads centrifugally from the mouth to the face and later to the extremities.^[^ ^118^ ^]^

The lesions eventually cover the face, scalp, trunk, limbs, soles of the feet, hands (including nail beds), anus, and genitals (glans, scrotum, labia majora, and penile shaft).^[^ ^119^ ^]^ In MSM‐related cases, lesions were mostly concentrated in the genital area (73%).^[^ ^116^, ^120^ ^]^ Starting as a flat red spot, the lesion progresses to vesiculopapule or vesiculopustule, and then crust, accompanying hyperpigmentation due to excessive skin lesions.^[^ ^3^, ^121^ ^]^ Some patients may also present with respiratory damage, oral ulcers, conjunctivitis, eyelid edema, pharyngitis, dep abscesses, subxiphoid lesions, and inguinal lymph node ulcers. Patients also frequently present with neuropsychiatric symptoms, including emotional instability and depression.^[^ ^118^ ^]^

The morphological changes of the rash range from maculopapules (flat lesions at the bottom) to small blisters (liquid‐filled blisters), and finally to pustules. The histopathological features of skin disorders are hyperplasia. The balloon degeneration of keratinocytes is accompanied by intracytoplasmic inclusion bodies in pericardium, and there are vesicles and pustules in the dermis. Infiltration of lymphocytes and macrophages could be found in the edematous skin. Moreover, poxvirus particles can be observed in the keratinocytes.^[^ ^118^ ^]^

During the pustular phase of the rash, there may be further periods of fever accompanied by severe complications, such as vomiting, diarrhea, pneumonia, encephalitis, septicemia, episcleritis, and corneal infections that can lead to vision loss. It can also cause delayed healing of inguinal ulcers, deep tissue abscesses, conjunctivitis, painful thumbs due to sublingual lesions, pruritus, and contact dermatitis.^[^ ^114^ ^]^ These complications mainly occur in children under 8 years of age, pregnant women, or people with impaired immune function. However, the recurrence of these symptoms is rare.^[^ ^122^ ^]^ Each stage lasts for 1–2 days, and the rash tends to crust after 12 days. The patient becomes non‐infectious 2–4 weeks after symptom onset. Lymphadenopathy, pre‐rash fever, and slow lesion maturation are important diagnostic characteristics of Mpox.^[^ ^123^ ^]^ For a definitive diagnosis of MPXV infection, PCR testing of lesions or effusions is required.^[^ ^124^ ^]^

Sequelae include secondary scarring and commonly overlapping infections, such as secondary bacterial infections, respiratory distress, bronchopneumonia, gastrointestinal involvement, dehydration, and encephalitis.^[^ ^114^, ^117^ ^]^

In addition to clinical symptoms, the microscopic features of MPXV are highly characteristic. Virus isolation and electron microscopy have shown active replication of the virus in the lungs and tongue,^[^ ^125^ ^]^ accompanied by observable microscopic lesions.^[^ ^126^ ^]^

### 6.1 The difference between smallpox and Mpox

Compared with smallpox, Mpox usually typically presents with a milder course of symptoms, and most patients recover within a few weeks. Similar to smallpox, its mortality rate is higher in children and immunocompromised individuals. Chickenpox is not clearly associated with lymphadenopathy, and there is no significant fever. Lesions (characterized by a persistent itch) are diverse but almost invariably affect the scalp and oral mucosa.^[^ ^114^ ^]^

### 6.2 Mortality rate

The mortality rates of Mpox depend on the variant, with rates ranging from 1% to 10% for the Congo Basin clade and up to 3.6% for Clade II. These rates annually increase in children and immunocompromised patients. Mortality rates are higher in individuals unvaccinated for smallpox, reaching up to 15% in the corresponding pediatric population.^[^ ^119^, ^127^ ^]^

## 7 DIAGNOSIS

**FIGURE 5:**
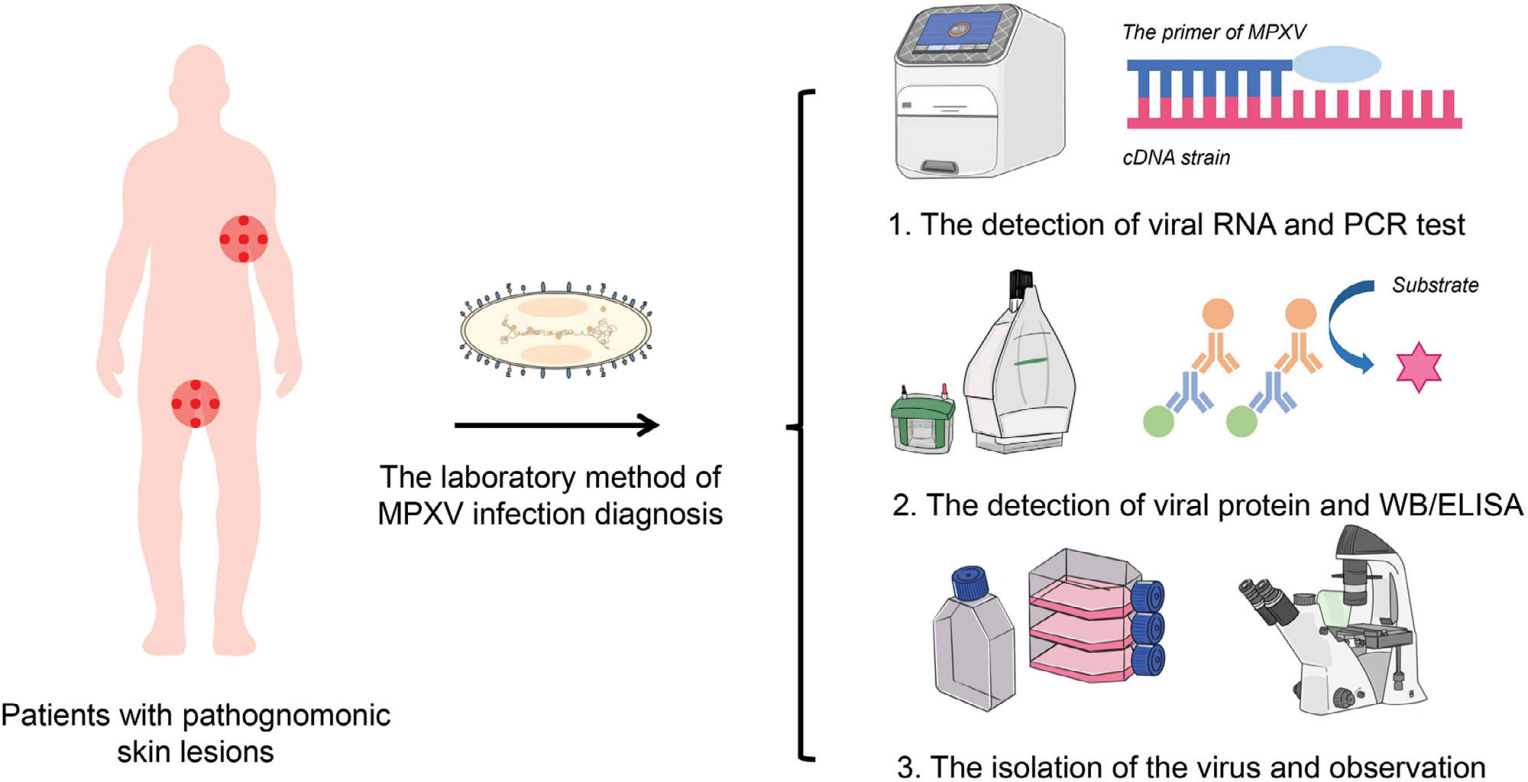
Main assays for the diagnosis of Mpox. The primary assays include nucleic acid detection, antigen and antibody detection, and pathological diagnosis. PCR has become a widely used and early tracing technique in diagnosing Mpox. Immunologic diagnosis depends on antigen specificity. Microscopic examination reveals simple pathogenic morphological differences.

Given the frequency of new cases in non‐endemic regions, assays for MPXV detection are constantly being updated. The current tests for MPXV infection are divided into nucleic acid, antigen, and antibody tests. For pathological diagnosis, among other laboratory tests, evaluation of environmental samples and identification by elimination of different orthopoxviruses are also crucial (Figure 5).

### 7.1 Nucleic acid assay

PCR is the main method for laboratory diagnosis because of its high sensitivity and validity,^[^ ^128^ ^]^ and is required for the confirmation of Mpox cases, as defined by the US Centers for Disease Control and Prevention (CDC) and WHO.^[^ ^129^ ^]^ The assay can be performed in two phases: pan‐orthopox PCR, followed by Mpox‐specific PCR.^[^ ^6^ ^]^ Clinical samples include saliva,^[^ ^130^ ^]^ blood,^[^ ^131^ ^]^ pustule swabs,^[^ ^57^ ^]^ vesicle swabs, and scabs/lesion crusts.^[^ ^132^ ^]^ MPXV is usually determined by the conserved sequence of the specific MPXV gene, including the orthopoxvirus DNA polymerase gene (E9L‐NVAR), envelope protein gene (B6R),^[^ ^133^ ^]^ DNA polymerase gene, dependent RNA polymerase subunit 18 (Rpo18), F3L gene,^[^ ^134^ ^]^ and the A‐type inclusion body gene (ATI gene).^[^ ^135^ ^]^ LC‐qPCR targeting the gene encoding the haemagglutinin (HA) protein was shown to specifically detect orthopoxviruses, enabling differentiation between various members of orthopoxviruses through melting curve analysis.^[^ ^136^ ^]^

In addition to PCR, several other nucleic acid detection techniques have been used to discriminate MPXV from other orthopoxviruses. Ryabinin et al.^[^ ^137^ ^]^ developed a microarray approach to simultaneously detect and identify six orthopoxviruses using specific oligonucleotide probes. Loparev et al.^[^ ^138^ ^]^ used restriction fragment length polymorphism (RFLP) assays to distinguish between multiple orthopoxviruses, including MPXV. Wolf et al.^[^ ^139^ ^]^ have used high‐throughput T cell receptor (TCR) sequencing to track and identify low‐frequency virus‐specific sequences. Loop‐mediated isothermal amplification (LAMP) technique^[^ ^140^ ^]^ and recombinase polymerase amplification (RPA) techniques^[^ ^141^ ^]^ can also diagnose MPXV quickly and efficiently while distinguishing between different Clades.

### 7.2 Antigen–antibody assays

Antigen and antibody assays also play an important role in the laboratory diagnosis of Mpox. In the late 20th century, scientists used radioimmunoassay (RIA) to detect antibodies in patient sera for smallpox, cowpox, and MPXV infections, providing a new method for the diagnosis of poxvirus‐related diseases.^[^ ^142^ ^]^

In the same century, electrophoretic techniques were widely used to identify orthopoxviruses. High‐resolution sodium dodecyl‐sulfate (SDS)‐sulfate‐polyacrylamide gel electrophoresis (SDS‐PAGE) was used to separate and compare the structural proteins of the variola virus, MPXV, and cowpox virus, allowing their effective identification.^[^ ^143^ ^]^

Enzyme‐linked immunosorbent assay (ELISA) is a common serological test that has been used to detect IgM and IgG in patients during the 2003 outbreak of Mpox in the USA and showed comparable results to those of PCR and viral cultures, indicating a high diagnostic performance for Mpox,^[^ ^144^ ^]^ but was not applied to confirm the infection.^[^ ^145^ ^]^ Nevertheless, the serological cross‐reactivity between orthopoxviruses and the possibility of false‐positive results from smallpox vaccines prevent the independent application of antigen and antibody assays to confirm MPXV infection.^[^ ^128^ ^]^ In 2016, Stern et al.^[^ ^146^ ^]^ established an antigen capture ELISA against the viral surface protein A27 that can detect all human pathogenic orthopoxviruses, including MPXV.

Monoclonal antibodies (mAbs) are important in protein detection. Researchers have developed an enzyme immunoassay (EIA) system based on mAbs for the species‐specific diagnosis of Mpox.^[^ ^147^ ^]^ The use of a combination of monoclonal capture antibodies that react with viral envelope epitopes and a polyclonal detection antibody in the ELISA improved the overall sensitivity of the assay.^[^ ^148^ ^]^ In 2022, a lateral flow assay based on four different MAbs was used to rapidly diagnose Mpox.^[^ ^149^ ^]^

### 7.3 Pathologic diagnosis

Pathological diagnosis is an adjunct to the laboratory diagnosis of Mpox and can be achieved by histology, immunohistochemistry, and microscopic examination of skin biopsy specimens such as scabs and infected tissues.^[^ ^150^, ^151^ ^]^ Using this method, MPXV and herpes simplex virus can be distinguished. But for varicella and other orthopoxviruses, PCR is required for confirmation.

### 7.4 Other assays

Rapid diagnosis of Mpox was developed in the early part of this century, along with the advancement of electron microscopy. Several new techniques have been developed recently. Fluorescent protein‐based reporter viruses can be used to explore the functions of these viruses.^[^ ^152^ ^]^ PET/CT‐based molecular imaging can be used to assess Mpox progression.^[^ ^153^ ^]^ Immunofiltration using ABICAP allows for the rapid and sensitive detection of orthopoxviruses.^[^ ^154^ ^]^

## 8 DRUG TREATMENT

Currently, no treatment for MPXV infections has been approved, and compounds effective in inhibiting MPXV infection are summarized in Table 1. And United States Strategic National Stockpile (SNS) recommends the clinicians to consider Tecovirimat, Brincidofovir, and Cidofovir (developed for use in patients with smallpox, Figure 1) as the options for the treatment of Mpox.

**TABLE 1:** Summary of compounds with efficacy against MPXV and potential mechanisms.

Compound	Animal/cellular/clinical trial results	Mechanisms	R&D team	Year	Ref.
PAV‐866	Has potent virucidal activity and can inhibit VACV infection before, during, or after virus adsorption; also effective against MPXV	Inactivates the viroid prior to infection, thereby inhibiting virus binding, fusion, and entry	Priyamvada et al.	2021	[223]
PA104	Inhibits 90% of virus transmission at 1.3 µm with a high selectivity index	Inhibits EV particle formation	Priyamvada et al.	2020	[224]
Retro‐2	Retro‐2 almost completely inhibited the spread of VACV as well as the closely related monkeypox virus between concentrations of 25 and 50 µm	Inhibits the retrograde pathway, preventing the virus from blocking the wrapping of the virosome with an additional double‐membrane envelope that allows microtubule transport, cytosolic split, and actin polymerization, thus inhibiting cell entry	Americo et al.	2016	[225]
Adamantane derivatives	The IC_50_ concentrations for inhibition of viral replication ranged between 0.133 and 0.515 µm	Inhibits viral particle replication by binding to the P37 protein target of poxviruses	Shiryaev et al.	2021	[226]
Tecovirimat	The drug is tolerable, safe, and effective in preventing disease and reducing mortality	Inhibits the formation of the extracellular enveloped virus necessary for cell‐to‐cell transmission	Siegrist EA et al.	NA	[227]
Brincidofovir	Effective and higher selection index than Cidofovir; it is not nephrotoxic but causes elevated liver enzyme levels	Inhibits viral DNA polymerase, thereby inhibiting viral DNA synthesis and replication	NA	NA	[8, 20, 168]
Cidofovir	Effective but can be nephrotoxic	Pyrimidines and purine analogs used as raw materials for DNA synthesis interfere with the replication of DNA viruses and prevent replication	Gilead	1995	[161]
Mitoxantrone	Improves the survival rate as well as the survival time of mice infected with orthopoxvirus; the drug showed strong synergy with Cidofovir	Inhibits viral DNA synthesis and replication	Altmann SE et al.	2012	[228]
Ribavirin and thiazofuran	Inhibits the replication of MPXV with relatively high sensitivity	Inhibits IMP, thereby blocking the synthesis of viral nucleic acids	E De Clercq	2001	[229]
Resveratrol	Significantly reduces the replication of MPXV	Suppresses subsequent expression by inhibiting the synthesis of viral DNA	Cao et al.	2017	[230]
5‐iodo‐2′‐deoxyuridine (IDU)	Effective in delaying poxvirus‐induced mortality	Inhibits poxvirus replication and inhibits viral DNA synthesis in a dose‐dependent manner	Neyts et al.	2002	[173]
*N*‐aminomethyl‐hemoglobin‐β‐aminothiourea	Effective and more selective	Inhibits transcriptional elongation, resulting in longer transcripts after termination of viral replication, and the increased intracellular concentration of dsRNA stimulates the cellular dsRNA‐dependent 2–5A pathway and establishes an antiviral state	Pirrung et al.	2005	[231]
3‐Deaza‐3‐fluoroaristomycin and its 5′ analogues	NA	Inhibits S‐adenosine homocysteine hydrolase (AdoHcy); affects DNA viruses	Chen et al.	2014	[232]
Pyridopyrimidinone inhibitors	NA	Blocks intermediate and late gene expression after viral replication, which can suppress the late stages of infection and persist after drug clearance	Dower et al.	2012	[233]
Carboxylic acid ion carrier nigericin	Highly selective with inhibitory activity against the cowpox virus	Moderate inhibition of transcription and translation of early cowpox genes, affecting viral DNA replication and mid‐ to late‐stage gene expression	Myskiw	2010	[234]
Interferon‐β	Human IFN‐β treatment significantly reduces MPXV production and transmission in vitro	IFN‐β induces expression of the antiviral protein MxA in infected cells, which in turn inhibits MPXV infection	Johnston et al.	2012	[235]
Kinase inhibitors (imatinib mesylate, dasatinib)	Limits virus transmission and has immunosuppressive effects	Blocks the transmission of poxvirus from infected cells	NA	NA	[17]
5‐azauracil	MpUNG protein is strongly inhibited and can be used as a therapeutic agent	Inhibition of the DNA repair protein uracil‐DNA glycosylase (UNG) blocks poxvirus pathogenesis	Duraffour et al.	2007	[171]
Recombinant immunoglobulin (rVIG)	Reduces morbidity, provides effective protection, is well tolerated, and reduces serum viral DNA levels	NA	NA	NA	[236]
Sarracenia	Effective in inhibiting poxvirus replication	NA	Arndt et al.	2012	[237]
SiRNA (siD5R‐2, siG7L‐1)	Significantly reduces the replication of MPXV	NA	Vigne et al.	2009	[238]
1,3‐Thiazolidin‐4‐one and thiazole derivatives	Good inhibitory activity, but exhibits moderate cytotoxicity	NA	Sokolova et al.	2018	[239]
5‐Bromodeoxyuridine	Hinders the replication of MPXV and cowpox virus	NA	Yoshii et al.	1978	[240]
D‐ and l‐2‐cyclopentenone	Has significant anti‐MPXV activity	NA	Jin et al.	2003	[241]
KAY‐2‐41	Protective effect, in vitro against VACV, CPXV, and CMLV	NA	Delaune and Iseni	2020	[20]
Mycophenolic acid	Shows some efficacy in vitro	NA	NA	NA	[159]
mDEF201 (an adenoviral vector interferon)	Delays death in cowpox virus‐infected mice	NA	Smee et al.	2011	[242]
*N*‐Methanocarbathymidine	Shows therapeutic effects in infected mice	NA	Delaune and Iseni	2020	[20]
NIOCH‐14	Oral administration reduces symptoms in mice infected with orthopoxvirus	NA	Mazurkov et al.	2016	[172]
*N*‐methylisatin 3‐thiosemicarbazone	Interferes with pox virus but causes nausea (used for smallpox)	NA	Bauer et al.	1963	[230]
4′‐Thiouridine	Delays death in cowpox virus‐infected mice	NA	Neyts et al.	2002	[173]
4′‐Thioisouridine derivative	Efficacy against CPXV, VACV, and strains of viruses resistant to Cidofovir or Tecovirimat	NA	Delaune and Iseni	2020	[20]
5′‐Homoaristeromycin	Strong antiviral activity against MPXV and cowpox virus	NA	Yang et al.	2005	[243]

### 8.1 Tecovirimat

Tecovirimat (4‐trifluoromethylphenol derivative, also called Tecovirimat or TPOXX) is a core protein cysteine protease inhibitor that achieves its antiviral effect by inhibiting the VP37 protein on the surface of orthopoxviruses.^[^ ^134^ ^]^ By inhibiting the production of extracellular viral particles (EVs) by binding to MPXV F13 (poxvirus phospholipase D, which plays a key role in the formation and infectivity of EVs), Tecovirimat prevents the maturation and release of viral particles from infected cells,^[^ ^155^ ^]^ thus blocking viral transmission between cells.^[^ ^156^ ^]^ The effectiveness of Tecovirimat has been demonstrated in preclinical trials, including four crucial studies in nonhuman primates indicating that the medication offered 95% protection against mortality, in contrast to a placebo. Trials in Phases 1 and 2 have examined the safety and adverse effect profile of Tecovirimat in human subjects.^[^ ^157^ ^]^ Tecovirimat has been used to treat systemic orthopoxvirus infection after rash onset. Based on changes in viral load, number of lesions, and survival in animal models, it has been shown to be highly effective in preventing disease and reducing mortality.^[^ ^158^ ^]^ In addition, human clinical trials have shown the drug to be tolerable and safe.^[^ ^20^ ^]^

In the US and Europe, Tecovirimat has been approved as an antiviral agent against orthopoxviruses such as MPXV, variola virus, and cowpox virus, and can significantly reduce Mpox mortality rates and the risk of virus transmission to susceptible populations.^[^ ^159^, ^160^ ^]^ It is currently one choice for treating of adults infected with MPXV (and children weighing more than 13 kg), and is ingested orally at a dose of 600 mg every 12 h (200 mg for a body weight of 13−25 kg and 400 mg for 25−40 kg) over a period of 14 days. Common side effects of taking Tecovirimat include headache (which may affect more than 1 in 10 people) and nausea (which may affect up to 1 in 10 people).^[^ ^159^ ^]^ Notably, Tecovirimat failed to reduce the duration of Mpox lesions among patients with clade I Mpox in the DRC, but the overall mortality among enrollees, regardless of whether they received the drug or not, was much lower than the mortality reported in the DRC (1.7% vs 3.6%) [https://www.nih.gov/news‐events/news‐releases/antiviral‐tecovirimat‐safe‐did‐not‐improve‐clade‐i‐mpox‐resolution‐democratic‐republic‐congo].

### 8.2 Cidofovir

Cidofovir (CDV, [(S)−1‐(3‐hydroxy‐2‐phosphono‐methoxypropyl) cytosine]) is a cyclic phosphonate nucleoside, a class of anti‐DNA viral drugs that act as pyrimidine and purine analogs for DNA synthesis and interfere with DNA virus replication.^[^ ^161^ ^]^ Cidofovir exhibits broad‐spectrum activity against most DNA viruses, with significant inhibition of poxviruses.^[^ ^162^, ^163^ ^]^ The drug works against MPXV by blocking viral DNA replication through the phosphonic acid moiety of Cidofovir.^[^ ^121^, ^164^ ^]^ However, because of the larger negative ionic group of the phosphonic acid moiety, the drug is prone to nephrotoxicity and can even cause irreversible kidney damage.^[^ ^165^ ^]^

Among antivirals against orthopoxviruses, Cidofovir has great potential for short‐term prophylaxis. Cidofovir is highly effective for treating VACV infections in SCID mice.^[^ ^162^ ^]^ According to the available data, 5% topical Cidofovir is effective in reducing viral titers in the skin, lungs, kidneys, and spleen, with some protection against systemic infections.^[^ ^166^ ^]^ However, during IV administration, hydration and probenecid treatment should be combined to reduce nephrotoxicity.^[^ ^121^ ^]^

### 8.3 Brincidofovir

Brincidofovir (CMX001; BCV) is the second drug approved by the US Food and Drug Administration (FDA) in 2021 for the treatment of smallpox.^[^ ^167^ ^]^ Brincidofovir is a lipid‐doped derivative of Cidofovir that is more effective in the treatment of poxvirus.^[^ ^20^, ^168^ ^]^ Brincidofovir inhibits viral DNA polymerase, thus inhibiting DNA synthesis and viral replication.^[^ ^8^ ^]^ In addition, increased cellular uptake of Brincidofovir allows for better conversion to its active form via intracellular enzymes, which contributes to its superior efficacy.^[^ ^169^ ^]^

The effectiveness of this drug in enhancing survival following infection has been demonstrated in both mice and rabbits. Its safety in human subjects was evaluated during clinical trials for cytomegalovirus disease in individuals receiving hematopoietic stem‐cell transplants. Brincidofovir has negative effects on the gastrointestinal and hepatic systems, and its safety profile is inferior to that of Tecovirimat.^[^ ^157^ ^]^ As Brincidofovir is known to serum transaminase and bilirubin levels, liver function tests should be performed before and during treatment for MPXV infection to avoid adverse outcomes.^[^ ^170^ ^]^

### 8.4 Other drugs

Many other compounds have shown promising results in the treatment of Mpox, that can be broadly classified as thioureas, nucleosides, nucleoside derivatives and nucleotide analogs, non‐nucleoside analogues, interferons, interferon inducers, and kinase inhibitors, among other unrelated compounds. Among these, 5‐azauracil, NIOCH‐14, and 4′‐thiouridine have shown good therapeutic activity.^[^ ^171^, ^172^, ^173^ ^]^ Nevertheless, further screening and modifications are required to improve safety and efficacy.

## 9 VACCINATION

Although there is currently no specific vaccine against MPXV, the CDC recommends administering a smallpox vaccine as a precautionary measure to prevent MPXV transmission. However, the efficacy of vaccinations remains uncertain. To prevent the virus spread and control the epidemic, it is imperative to promptly vaccinate high‐risk populations, including MSM,^[^ ^174^ ^]^ healthcare workers, and occupationally exposed individuals (e.g. veterinarians).

The CDC recommends that individuals exposed to MPXV and those who may be more susceptible should be vaccinated. Can voluntary vaccination eradicate MPXV in fully endemic, disease‐free, and previously neglected semi endemic cases? The results of a related study showed eradication by vaccination in a semi‐endemic equilibrium, but not in a fully endemic equilibrium.^[^ ^175^ ^]^ Studies have also shown that changes in neutralizing antibody titers following smallpox vaccination are associated with sex.^[^ ^175^ ^]^ The UKHSA reported that it would provide smallpox vaccines (Imvanex) to MSM to control the recent Mpox outbreak (https://www.gov.uk/guidance/monkeypox‐outbreak‐vaccination‐strategy). The Advisory Committee on Immunization Practices (ACIP) recommends ACAM2000 vaccination for laboratory workers, persons at risk of occupational exposure, and healthcare workers.^[^ ^176^ ^]^

**FIGURE 6:**
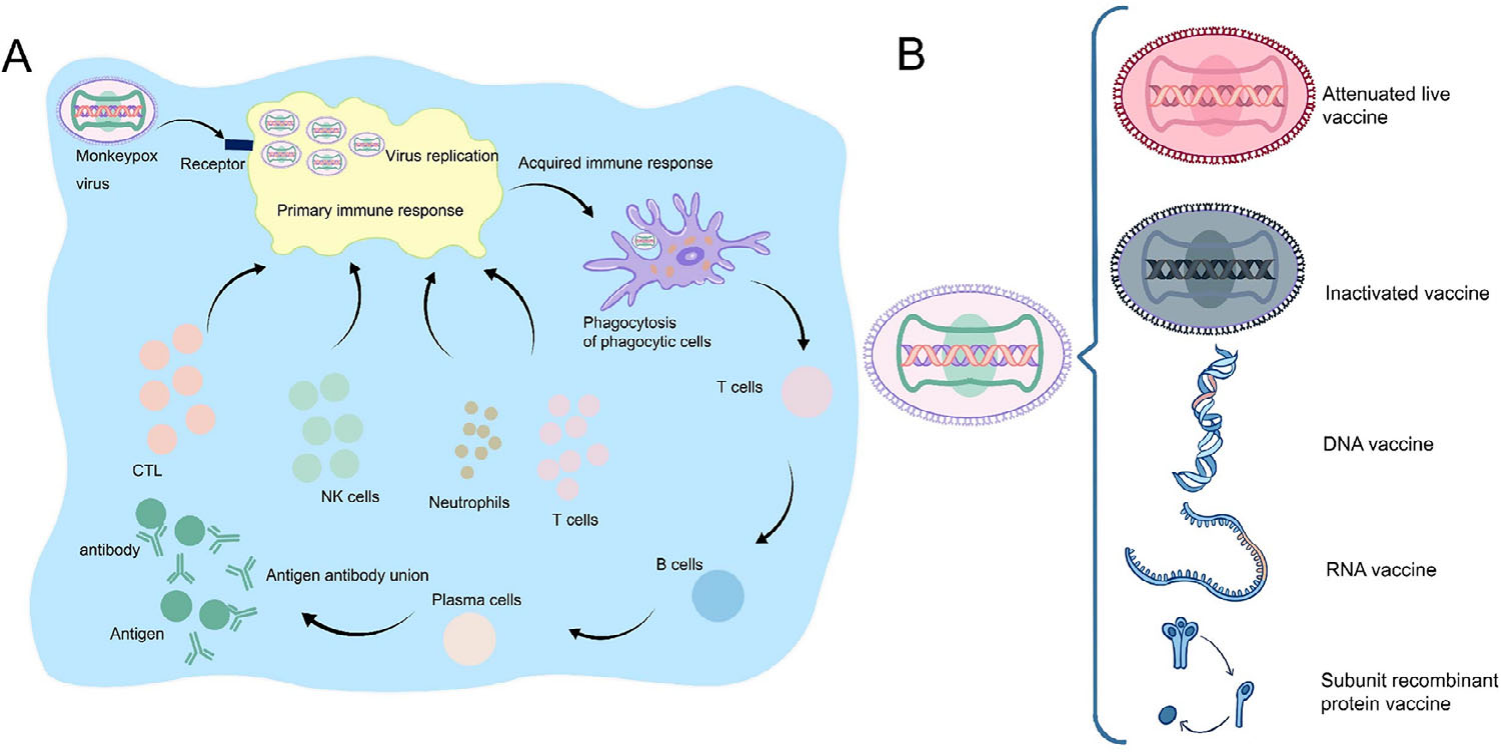
Vaccine development and immune response pathways for prevention of MPXV. Vaccine development and immune response pathways for MPXV prevention. (A) Immune response pathways for vaccines against MPXV. The virus recognizes the receptor and binds to the receptor; CTL, NK cells, neutrophils, T cells, etc. accumulate around the infected cells and activate the primary immune pathway in the body to inhibit viral replication; macrophages phagocytose the virus and initiate the adaptive immune response, B cells differentiate and proliferate to plasma cells that can produce antibodies, plasma cells secrete a large number of antibody molecules into the blood circulation, antibodies bind to antigens to form antigen complexes, and further inactivate and remove antigens. (B) MPXV vaccines are mainly used to prevent viral infections. Common vaccines include live‐attenuated, inactivated, DNA, RNA, and subunit recombinant protein vaccines.

The smallpox vaccine provides a robust cross‐protective immune response against variola virus and MPXV by stimulating T‐ and B‐cell activities^[^ ^177^ ^]^ (Figure 6A). Notably, B‐cell depletion abolishes the protective effect of the vaccine.^[^ ^178^ ^]^ Helper CD4^+^ T cells are required for the induction of adaptive CD8^+^ T and B cell responses. In immunodeficient macaques with CD4^+^ T cell counts < 300 cells/mm^3^, smallpox vaccination is ineffective against lethal MPXV infections.^[^ ^179^ ^]^ CD4^+^ T cells recognize HLA‐DR1‐restricted epitopes on the conserved VACV proteins A24R and D1R, which can be exploited to analyze CD4^+^ T cell responses to existing or next‐generation smallpox vaccines.^[^ ^180^ ^]^ Following infection with MPXV, smallpox vaccine‐protective E3L‐specific T‐cells protect patients from viral attack by killing infected cells and peptide‐loaded target cells.^[^ ^181^ ^]^ D8 is a protective antigen against intracellular maturation. The addition of the D8 antigen, monovalent and polyvalent poxvirus vaccines resulted in a strong protective antibody response and significantly improved vaccine efficacy.^[^ ^182^ ^]^

### 9.1 Smallpox vaccine ACAM2000

Clinical trials have demonstrated that the cowpox strain DryVax may cause serious adverse reactions; thus, clinical trials were discontinued.^[^ ^183^ ^]^ In August 2007, the second‐generation smallpox vaccine ACAM2000 officially replaced the orthopoxvirus vaccine DryVax and was approved by the FDA for active immunization of people over 18 years of age who are at a high risk of smallpox infection^[^ ^184^ ^]^ (Figure 6B). The CDC recommended vaccination with ACAM2000 during the latest Mpox outbreak. The first dose of the ACAM2000 vaccine within 4 days of exposure to MPXV should protect against the virus, whereas the same dose within 4−14 days may reduce clinical symptoms, but not prevent the onset of disease.^[^ ^170^ ^]^

ACAM2000 is a replication‐competent poxvirus that can cause skin reactions at vaccination sites. In contrast, MVA‐BN, the modified vaccinia Ankara‐Bavarian Nordic (MVA‐BN strain), showed no skin reactions at the site of vaccination. The CDC recommends that immunosuppressed individuals avoid using ACAM2000, which may have serious adverse reactions, such as pericarditis and post‐vaccination encephalitis.^[^ ^170^ ^]^

### 9.2 Smallpox vaccine MVA‐BN

MVA‐BN (also known as JYNNEOS, IMVAMUNE, or Imvanex) is a non‐replicating live attenuated cowpox virus vaccine. MVA‐BN was approved by the FDA in 2019. In 2013, MVA‐BN was authorized by the European Drug Agency for safe use in immunodeficient patients.^[^ ^174^ ^]^ In November 2021, MVA‐BN was officially launched as an alternative to ACAM2000 for primary vaccination and booster doses, particularly for Mpox prophylaxis.^[^ ^185^ ^]^ In August 2022, the FDA authorized a third‐generation MVA‐BN vaccine that could be administered subcutaneously at a lower dose to achieve an appropriate immune response. MVA‐BN is a two‐dose vaccine that requires 14 days after the second dose for maximal immune protection. The first dose of MVA‐BN vaccination is thought to produce a strong immune response within the first two weeks and may reduce clinical symptoms and disease severity (https://www.gov.uk/guidance/monkeypox‐outbreak‐vaccination‐strategy). The WHO recommends vaccination within 4 days of exposure.^[^ ^15^ ^]^ MVA‐BN is used either as pre‐exposure prophylaxis (PrEP) or post‐exposure prophylaxis (PEP) to prevent MPXV infection, especially in individuals with high‐risk sexual behavior, which is needed to control ongoing outbreaks of the third branch of the MPXV.^[^ ^15^ ^]^ Vaccination with MVA‐BN is considered effective in improving the safety and immunogenicity among healthcare workers.^[^ ^186^ ^]^ Recombinant MVA provides approximately 2.7 years of protection against diseases caused by immunodeficiency viruses and long‐term immune protection against orthopoxviruses in non‐human primates.^[^ ^186^, ^187^ ^]^ Additionally, MVA‐BN can produce a protective immune response against a wide range of orthopoxviruses, with 85% protection against MPXV.^[^ ^184^ ^]^ Studies have reported no adverse reactions in pregnant women or in animal models. Furthermore, the safety of the MVA‐BN vaccine was confirmed in both the MVA85A TB vaccine trial in infants and the malaria vaccine candidate trial.^[^ ^188^, ^189^ ^]^ Although it is unclear whether MVA‐BN is passed through breast milk, it is considered safe for breastfeeding.^[^ ^91^ ^]^

### 9.3 Smallpox vaccine LC16m8

LC16m8 has been licensed as a smallpox vaccine in Japan. The LC16m8 vaccine is a highly attenuated poxvirus vaccine that lacks the expression of the membrane protein B5R and effectively protects non‐human primates from MPXV.^[^ ^190^, ^191^ ^]^ The safety of the vaccine depends on T cell response. In rhesus macaques depleted of T or B cells, the LC16m8 vaccination had no adverse effects, whereas rhesus macaques vaccinated with Dryvax developed progressive cowpox, and B cell depletion did not affect the adverse effects induced by either vaccine. Therefore, LC16m8 is a safer and more effective alternative to the ACAM2000 and DryVax vaccines in immunocompromised individuals.^[^ ^192^ ^]^

### 9.4 Recombinant vaccine against MPXV

Recombinant vaccines prevent MPXV in animal models and produce different antibody neutralization responses. Hooper et al.^[^ ^193^ ^]^ first demonstrated the effectiveness of subunit vaccines against variola virus and MPXV using a DNA vaccine consisting of four poxvirus genes (L1R, A27L, A33R, and B5R) that protected non‐human primates, rabbits, and mice from orthopoxvirus.^[^ ^194^, ^195^, ^196^ ^]^ Subunit vaccination with aluminum hydroxide and CpG as adjuvant provided a more uniform antibody response and a stronger IgG1 response.^[^ ^197^, ^198^ ^]^ Adjuvants of recombinant proteins (QS‐21 and, to a lesser extent, alum+CpG oligodeoxynucleotides) enhanced orthopoxvirus antibody neutralization responses in infected mice, and macaques vaccinated with recombinant poxvirus proteins and QS‐21 produced neutralizing antibodies against MPXV. Viral load and disease severity were reduced in macaques compared to those in the unvaccinated control group.^[^ ^199^ ^]^

Hooper et al.^[^ ^200^ ^]^ produced antibody‐neutralizing responses in mice and non‐human primates (crab‐eating macaques) using a smallpox vaccine with virus‐like replicon particles (VRPs) of VACV (including *A33R*, *B5R*, *A27L*, and *L1R* genes) delivered via alphavirus replicons, which significantly inhibited the transmission of the smallpox virus. The single‐boost VRP smallpox vaccine is expected to be an effective alternative to the live cowpox viral smallpox vaccine.

### 9.5 Combination of smallpox vaccines and drugs

The National Institute of Allergy and Infectious Disease supports studies in non‐human primates to determine the effectiveness of vaccination in conjunction with smallpox treatment with Tecovirimat. A previous study found that the immune response induced by the MVA‐BN or ACAM2000 smallpox vaccine was not significantly affected by the concomitant use of Tecovirimat. Evaluation of the effectiveness of vaccine–drug combinations in a murine pox model showed that vaccination with DryVax or ACAM2000 combined with the antiviral drug Brincidofovir (CMX001) effectively reduced the adverse effects associated with the vaccine.^[^ ^201^ ^]^ Although the combination of a single dose of Cidofovir and DryVax reduces the adverse effects of vaccination, it interferes with vaccine‐induced immune responses, including antibody and helper T‐cell responses.^[^ ^202^ ^]^ When the neutralizing activity of antibodies against the vaccine and the efficacy of protection against MPXV attacks in non‐human primates were assessed using a combination of TPOXX and ACAM2000 smallpox vaccines, all 12 animals in the vaccinated/placebo group survived, and 12 of the 13 animals in the vaccinated/TPOXX group survived after MPXV infection. Significant clinical symptoms were observed in the vaccinated/TPOXX group compared with the vaccinated/reassurance group, suggesting that the antibody neutralization response and immunogenicity of the vaccine may be affected by the combination of the vaccine and drug administration.^[^ ^203^ ^]^ A combination of Tecovirimat and DryVax did not reduce antibody neutralization titers or impair vaccine‐induced cytoprotective immunity, whereas Tecovirimat co‐administered with the vaccine provided equivalent short‐ and long‐term protection against lethal intranasal viral doses in vv‐wr mice compared to vaccination alone.^[^ ^204^ ^]^

## 10 CONCLUSION AND PERSPECTIVE

The 2022 outbreak of Mpox signifies the transition of Mpox from a regional disease to a global concern. In light of the COVID‐19 pandemic, it is evident that monkeypox outbreaks are likely to become more frequent in the future, necessitating proactive measures by all stakeholders to mitigate the public health burden, particularly among immunocompromised populations.^[^ ^205^ ^]^ In addition, comprehensive characterization of MPXV's whole genome and epidemiology is imperative for optimizing prevention strategies based on pathogen evolution and its adaptation to vectors and humans.^[^ ^117^, ^206^ ^]^

Understanding how viral pathogens invade the host cell is the key to the development of antiviral agents. As our understanding of these essential virus–host interactions deepens, we may have the opportunity to specifically target them, leading to innovative intervention strategies. Additionally, studying viral genomics enables a comprehensive analysis of the MPXV infection process and the elucidation of relevant therapeutic targets.

In 2022 alone, the number of Mpox cases diagnosed worldwide exceeds the cumulative reported cases from the first recorded case in 1970 until the end of 2021. Three hypotheses were proposed to elucidate this phenomenon: first, genetic drift within the virus itself, second, increased animal‐human exposure, and third, a potential increase in Mpox incidence due to discontinuation of smallpox vaccination campaigns.^[^ ^1^, ^207^, ^208^ ^]^ Notably, intimate contact appears to be a particularly efficient mode of MPXV transmission, especially among MSM individuals.^[^ ^11^, ^14^, ^209^ ^]^ Given the high prevalence of MPXV infections in HIV‐positive patients, APOBEC3‐driven specific mutations may contribute to reduced pathogenicity and symptoms associated with MPXV infection, while the transmission ability of MPXV increased.^[^ ^209^ ^]^ These findings indicate that the spread of MPXV in MSM, particularly those with HIV infection, led to adaptive viral evolution, resulting in biased mutations and the acquisition of abnormal viral characteristics, such as increased transmission and decreased virulence. Furthermore, it should be noted that the majority of newly diagnosed patients do not possess a documented travel history to Africa or any known exposure to potentially infected animals,^[^ ^210^ ^]^ implying that MPXV infection can be acquired by close contact with confirmed cases. Fortunately, the prevalence of infection among the general population remains low.

Inoculation serves as an efficacious strategy for defending viral infections, and JYNNEOS is currently employed as PrEP and PEP to prevent MPXV infection. However, there are concerns regarding the effectiveness and undersupply of this vaccine. A study conducted on unvaccinated healthy individuals against smallpox revealed that neither one nor two doses of JYNNEOS resulted in a substantial increase in neutralizing antibodies against MPXV.^[^ ^211^ ^]^ A clinical trial with 524 enrolled individuals showed that intradermal (ID) vaccination (1/5 standard dose) achieved rates of induced neutralizing antibody production comparable to those of subcutaneous (SC) vaccination.^[^ ^212^ ^]^ According to FDA recommendations, the administration of the JYNNEOS vaccine should consist of two doses given at 28‐day intervals for subcutaneous (SC) vaccination or two doses at 28‐day intervals with each dose being 0.1 mL (equivalent to 1/5th of the standard dose) for intradermal (ID) vaccination (https://www.fda.gov/media/160774/download).

Previously, MPXV infections were confined to regions of Africa, but the surging Mpox cases in non‐endemic areas led to a worldwide concern, burdening the medical budget and causing racist stereotypes and stigmatizations. Previously, MPXV infections were predominantly limited to regions in Africa, but the surging cases of Mpox in non‐endemic areas have raised global concerns, imposing significant strain on healthcare budgets. To prevent the spread of Mpox, it is crucial to provide affordable vaccines to regions with high prevalence rates and targeted populations especially those with HIV infection.^[^ ^213^ ^]^ Furthermore, it is imperative to ensure the provision of vaccines for healthcare professionals, veterinarians, and associated staff.^[^ ^214^, ^215^ ^]^ Until a new vaccine is available, discontinuing JYNNEOS for high‐risk populations poses a greater risk than MPXV transmission. In addition, considering potential complications from vaccinating HIV‐infected patients and adverse effects on fetuses when vaccinating pregnant women, special attention should be given to the health status of vaccinated individuals with effective management of any complications.^[^ ^216^, ^217^ ^]^ A study conducted by Edghill‐Smith et al.,^[^ ^179^ ^]^ involving MPXV‐infected macaques with HIV infection, revealed an impairment in vaccinia‐specific immunoglobulin (Ig) switching from IgM to IgG. As CD4^+^ cells are targeted by HIV‐1 infection; vaccination strategies bypassing CD4^+^ cells are necessary. In addition, two FDA‐approved drugs, Brincidofovir and Tecovirimat, which were approved for smallpox treatment, demonstrated efficacy against MPXV infection in animal models. Although the efficiency specifically on Mpox patients lacks sufficient documentation, these two drugs were approved for emergency use due to their antiviral effectiveness observed in animal models.^[^ ^11^ ^]^

There is currently no vaccine for monkeypox available, possibly due to the relatively low infection rate of monkeypox, and companies lack sufficient motivation to develop relevant vaccines. Given the great success of mRNA vaccines in preventing COVID‐19 outbreaks,^[^ ^218^ ^]^ a series of MPXV vaccines have been developed by multiple teams, most are expressing viral protein A27L, A33R, L1R, and B5R in vivo, inducing related antibodies, and some exhibited significant efficacy in the animal models.^[^ ^219^, ^220^, ^221^, ^222^ ^]^ These MPXV vaccines and drugs under development can serve as potent tools in combating the Mpox epidemic.

## AUTHOR CONTRIBUTIONS

Huahao Fan, Renald Blundell, Yigang Tong, Lihua Song, Qingquan Liu, and Xiaolong Xu conceived the study. Lin Jiang, Ailan Xu, Lin Guan, Yong Tang, Guangshuai Chai, Junya Feng, and Yueqi Wu reviewed the literature and drafted the manuscript. Maochen Li improved the quality of figures. Xiaolong Xu, Qingquan Liu, Xiaojing Liu, and Chuxie Zhang conducted a thorough review and provided valuable feedback, making significant revisions to improve the quality of this manuscript. All the authors reviewed the manuscript.

## CONFLICT OF INTEREST STATEMENT

The authors declare no conflicts of interest.
